# Supplementary material for: Liquid Biopsy Targeting Monocarboxylate Transporter 1 on the Surface Membrane of Tumor-Derived Extracellular Vesicles from Synovial Sarcoma
Source: Cancers (Basel). 2021 Apr 11;13(8):1823. doi: 10.3390/cancers13081823 (PMC8069269; doi:10.3390/cancers13081823)
Supplement: Supplementary file 1 [file cancers-13-01823-s001.tgz › Supplementary Figure.pdf]

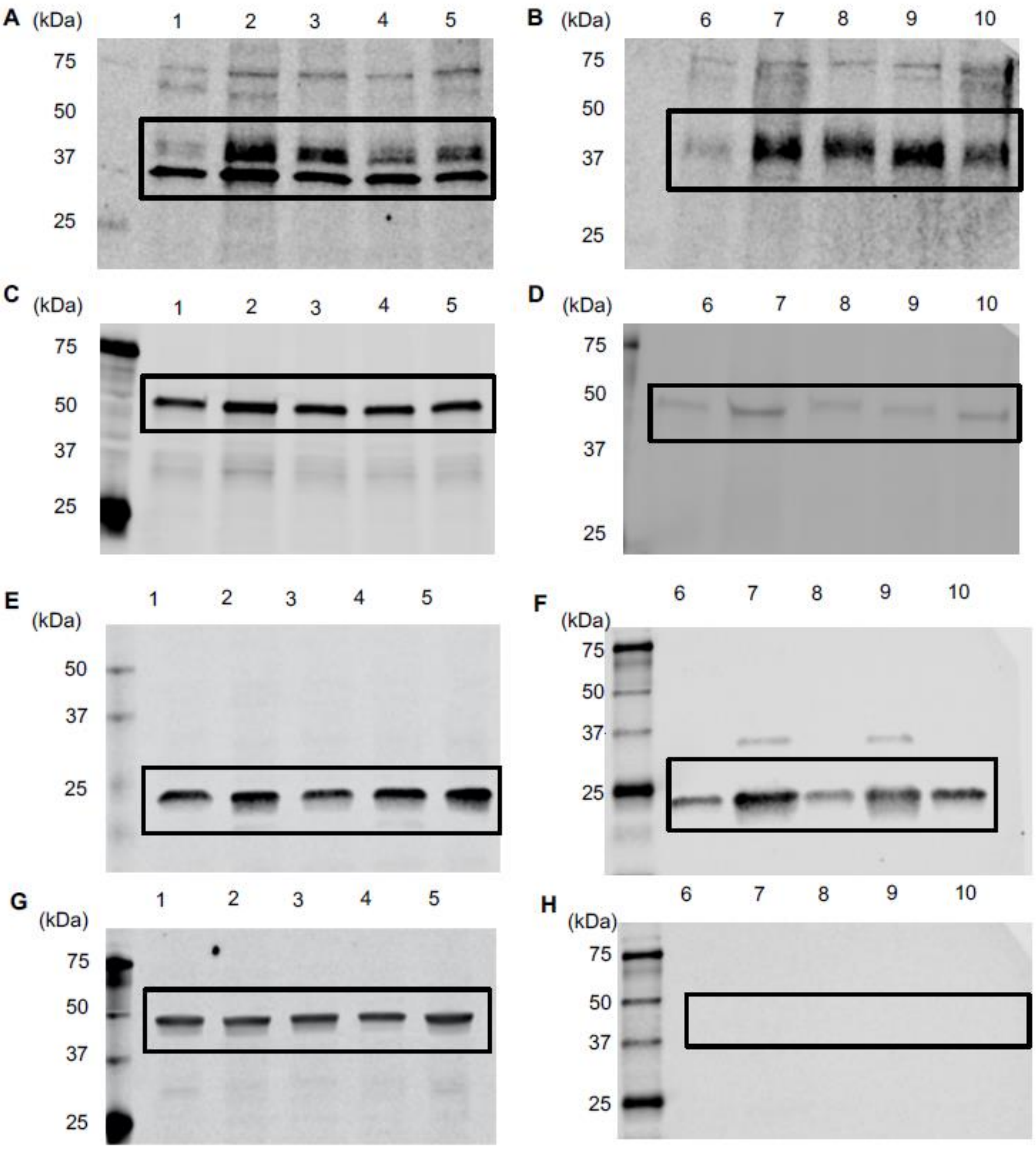

Lane No.	Type of samples	Cell lines	Lane No.	Type of samples	Cell lines
1	Cells	Aska-SS	6	EVs	Aska-SS
2	Cells	HS-SY-II	7	EVs	HS-SY-II
3	Cells	SYO-1	8	EVs	SYO-1
4	Cells	YaFuSS	9	EVs	YaFuSS
5	Cells	Yamato-SS	10	EVs	Yamato-SS

Supplementary Figure S1. Full-length blots shown in Figure 1C. (A, B) MCT1 (43kDa) and (C, D) Tubulin (50kDa). (E,F) CD81 (23kDa) and (C,D) Tubulin (50kDa).
